# Emergence agitation after intraoperative neurolytic celiac plexus block with alcohol: a case report

**DOI:** 10.1186/s12871-021-01426-2

**Published:** 2021-08-16

**Authors:** Huixuan Zhou, Yinbing Pan, Cunming Liu, Xiaodi Sun

**Affiliations:** Department of Anesthesiology and Perioperative Medicine, The First Affiliated Hospital of Nanjing Medical University, No. 300 Guangzhou Road, Nanjing, 210029 China

**Keywords:** Emergence agitation, Neurolytic celiac plexus block, Propofol, Acute alcohol intoxication, Case report

## Abstract

**Background:**

Emergence agitation after general anesthesia may cause several undesirable events in the clinic during patient anesthesia recovery, and acute alcohol intoxication, while rare in surgery, is one of the risk factors.

**Case presentation:**

A 66-year-old male patient was found to have pancreatic tail neoplasm upon computed tomography (CT) examination. The surgeon planned to resect the pancreatic tail under general anesthesia. However, the surgeon found extensive tumor metastasis in the abdominal cavity, and thus performed a neurolytic celiac plexus block (NCPB) with 40 ml 95% ethyl alcohol and finished the surgery in approximately 1 h. Twenty minutes later, the patient was extubated and developed significant emergence agitation in the postoperative care unit, characterized by restlessness, uncontrollable movements, confusion and disorientation. The patient was flushed and febrile with an alcohol smell in his breath and was unable to follow commands. Patient symptoms were suspected to be due to acute alcohol intoxication. Thus, the patient was given 40 mg of propofol intravenously. Following treatment, the patient recovered with less confusion and disorientation after approximately 10 min. After treatment with propofol twice more, he regained consciousness, was calm and cooperative, had no pain, and could obey instructions approximately 1 h and 40 min following the last treatment. Following this treatment, the patient was transferred to the inpatient ward and felt well.

**Conclusions:**

It is paramount to correctly identify the underlying cause of emergence agitation in order to successfully manage patient symptoms, since treatment plans vary between different etiological causes. Emergence agitation may be due to acute alcohol intoxication after intraoperative use of alcohol.

## Background

Emergence agitation after general anesthesia is an ongoing challenge in the clinic, and is characterized by confusion, disorientation, restlessness, and uncontrollable constant movement in the early phase of general anesthesia recovery [1]. It may cause several undesirable events for the patient, such as self-extubation, removal of catheters, wound dehiscence, fall injury, etc. The incidence of emergence agitation in adults ranges from 4.7 [2] to 21.3% [3] based on the individual situation, anesthesia technique, surgery type, duration time, etc. There are many risk factors for emergence agitation, one of which is acute alcohol intoxication [2, 4]. However, emergence agitation after immediate extubation caused by acute alcohol intoxication is rare. In this report, we present a case of significant emergence agitation after intraoperative neurolytic celiac plexus block with ethyl alcohol.

## Case presentation

A 66-year-old male patient had experienced epigastric pain for half a month. Upon CT examination, pancreatic tail neoplasm was observed. The surgeon planned to resect the pancreatic tail under general anesthesia. The patient developed hypertension and his electrocardiogram showed sinus rhythm and high voltage in the left ventricle. He weighed 66 kg and was American Society of Anesthesiologist classification II. Before anesthesia induction, his heart rate was 82 beats per minute, pulse oxygen saturation was 98%, invasive blood pressure was 140/85 mmHg and blood gas analysis was normal. We started general anesthesia induction at 08:45. With preoxygenation for 3 min, the patient was given 2 mg of midazolam, 12 mg of etomidate, 0.1 mg of fentanyl, and 12 mg of cisatracurium intravenously and sequentially. Two minutes later, the patient was given 160 µg of remifentanil [5] and was intubated with a 7.5 mm internal diameter tube. Before incision, the patient was given 0.4 mg of fentanyl. And the surgery started at 09:05. General anesthesia was maintained with propofol at 100 μg/kg/min, remifentanil at 0.1 μg/kg/min, cisatracurium at 1.5 μg/kg/min, dexmedetomidine at 0.005 μg/kg/min, and sevoflurane at 1–2%. Invasive systolic pressure ranged between 110 and 140 mmHg and diastolic pressure ranged between 60 and 70 mmHg. The patient’s invasive systolic pressure fell below 110 mmHg twice and was quickly restored. The patient’s heart rate ranged from 70 to 100 beats per minute. When the surgeon opened the patient’s abdominal cavity, he found extensive tumor metastasis and performed neurolytic celiac plexus block (NCPB) with 40 ml 95% ethyl alcohol at 09:20. The surgery lasted for approximately 1 h and ended at 10:10. Then the patient was transferred to the post-anesthetic care unit (PACU). Twenty minutes later, the patient was extubated and developed significant emergence agitation. He was confused, disoriented, flushed, febrile, unable to follow commands, and restless and had an alcohol smell in his breath. We suspected acute alcohol intoxication and administered 40 mg of the short-acting sedative propofol intravenously. Approximately 10 min later, he recovered with less confusion and disorientation. After treatment with propofol twice more, he regained consciousness and was calm, had no pain or urethral irritation, and could obey instructions approximately 1 h and 40 min following the final treatment. His invasive systolic pressure ranged between 90 and 120 mmHg and diastolic pressure between 40 and 60 mmHg in the post-operative phase. His heart rate ranged between 70 and 80 times per minute before extubation in PACU, was 130 times per minute at the moment of extubation and ranged between 70 and 90 times per minute after propofol treatment. His pulse oxygen saturation ranged between 94 and 100% on mask-based oxygen while between 89 and 92% on air. In the end, he was transferred to the inpatient ward and felt well.

## Discussion and conclusions

Identifying possible etiology of emergence agitation is paramount for successful management because the treatment of different etiologies is very different. Risk factors for emergence agitation include age, recent smoking, sevoflurane anesthesia, postoperative pain and the presence of a tracheal tube or urinary catheter [4]. Post-operative pain commonly causes emergence agitation after open abdominal surgery and the management is to administer enough analgesic drug to relieve patient discomfort. In this case, the open abdominal surgery lasted only 1 h, and the patient (weighing 66 kg) was given 0.5 mg of fentanyl in total. The analgesic drug dose was normal for such a patient in the clinic. Therefore, we did not attribute his agitation to post-operative pain following a comprehensive evaluation. In surgery, the patient underwent an NCPB with 40 ml 95% alcohol. The criteria of the Diagnostic and Statistical Manual of Mental Disorders (5th edition) for acute alcohol intoxication [6] include recent alcohol ingestion history, clinically significant maladaptive behavioral or psychological changes and some symptoms that develop during or shortly after alcohol use. Because the patient developed significant emergence agitation after alcohol use in the NCPB and was flushed and febrile with an alcohol smell in his breath, we suspected acute alcohol intoxication. The patient was then given propofol for sedation three times. Approximately 1 h and 40 min later, he regained consciousness and was calm and obeyed our instructions.

We asked the patient if he had incisional pain or urethral irritation that he denied having. Thus, we excluded post-operative pain and the urinary catheter from potential causes to his emergence agitation. We speculated that the patient ingested a large amount of alcohol during the NCPB through the rich vascular network around the celiac plexus. We clinically diagnosed his mental disorder as acute alcohol intoxication according to the noted criteria of the Diagnostic and Statistical Manual of Mental Disorders for acute alcohol intoxication. The degree of acute alcohol intoxication is judged by two methods: clinical judgement and blood alcohol level test [7]. Based on the clinical assessment, we diagnosed that the patient belonged to a moderate level, indicated by his confusion, disorientation, and lost motor coordination according to the clinical degree category of acute alcohol intoxication [8]. For a patient with emergence agitation at such a high degree, sedation with propofol was effective and dexmedetomidine could also be a first-line pharmacological agent for treatment [9]. For severe cases, metadoxine and naloxone are preferred. Metadoxine accelerates ethanol metabolism, which accelerates patient recovery from intoxication and improves the behavioral toxic symptomatology [8]. Naloxone reduces β-endorphin in the brain, reduces central inhibition of alcohol, and shortens the time for recovery of consciousness [10], however, it may cause severe incision pain and therefore aggravate agitation for postoperative patients. Thus, the anesthetist should weigh the advantages and disadvantages before using naloxone. Tei A [11] reported a case of acute alcohol intoxication after ethanol fixation for an ovarian chocolate cyst in 1996. Alcohol is used in some surgeries, such as ethanol fixation for ovarian chocolate cyst or NCPB. However, since these procedures are not very common in the clinic, anesthetists are often unaware of the potential that surgery-based alcohol use could be the underlying cause of patient symptoms when dealing with a patient with delirium, especially when the anesthetists are unaware of the alcohol use during surgery. We reported this case to remind anesthetists and surgeons of acute alcohol intoxication when patients experience delirium after the use of alcohol. They can administer different medications according to the severity of patient delirium. In this case, the patient experienced moderate delirium and was able to recover with only propofol sedation without hypotension, which is a common complication of NCPB after sympathetic block and typically leads to the need for continuous fluid support and catecholamines infusion until the patient is stable.

There are some limitations in the case. First, we did not test the blood alcohol level because we could not do the test in our hospital. So we suspected acute alcohol intoxication according to clinical judgement not the blood alcohol level [7]. Second, we did not do blood gas analysis during the treatment process although he was transferred to the inpatient ward and felt well. This is the first time we met acute alcohol intoxication patient in the operation.

In conclusion, it is paramount for successful management of emergence agitation to identify the possible underlying etiology. Emergence agitation after intraoperative use of alcohol may attribute to acute alcohol intoxication.

## Data Availability

The raw data presented in this article could be searched in medical system of the First Affiliated Hospital of Nanjing Medical University. All data and materials described in the manuscript will be freely available to any scientist who wants to use them for non-commercial purposes.

## Abbreviation

NCPB: Neurolytic celiac plexus block
